# How Power from the Network Is Associated with Post-Traumatic Growth During COVID-19: The Mediating Roles of Gratitude and Cognitive Reappraisal

**DOI:** 10.3390/bs15030335

**Published:** 2025-03-10

**Authors:** Xiangxing Hao, Yimeng Cui, Mingyu Zhao, Yanling Chen, Zhi Ren, Lin Zhang

**Affiliations:** 1 Department and Institute of Psychology, Ningbo University, No. 818 Fenghua Road, Ningbo 315211, China; haoxiangxing2023@163.com (X.H.); zhaomingyu11@qit.edu.cn (M.Z.); 2 School of Psychology, South China Normal University, No. 55 West Zhongshan Avenue, Tianhe District, Guangzhou 510631, China; dawangyuzhou7@gmail.com; 3 Department of Psychology, Renmin University of China, No. 59 Zhongguancun Street, Haidian District, Beijing 100872, China; chenyanling7100@163.com

**Keywords:** online social support, gratitude, cognitive reappraisal, post-traumatic growth

## Abstract

Background: The COVID-19 pandemic has posed significant psychological challenges worldwide, but individuals may also experience post-traumatic growth (PTG)—positive psychological changes following adversity. Identifying factors associated with PTG during global public health crises is crucial for advancing trauma recovery research and informing evidence-based interventions. As interpersonal interactions increasingly move to virtual platforms, online social support has become a key source of psychological resilience. Yet, how online social support facilitates PTG remains unclear, especially in large-scale adversities, like the COVID-19 pandemic. Objective: This study investigates the association between online social support and post-traumatic growth (PTG) in the context of the COVID-19 pandemic and examines the mediating roles of gratitude and positive reappraisal. Method: A cross-sectional online survey was conducted in March 2022, recruiting 556 college students (70.83% female). Online social support, PTG, gratitude, and cognitive reappraisal were assessed using validated tools, including the College Students’ Online Social Support Testing Questionnaire, Post-Traumatic Growth Inventory (PTGI), Gratitude Adjective Checklist, and Cognitive Emotion Regulation Questionnaire (CERQ). Results: Participants reported moderate PTG levels. Online social support was positively associated with PTG and exerted both direct and indirect effects. The indirect effects occurred through a sequential mediation pathway, wherein online social support first enhanced gratitude, which in turn, promoted cognitive reappraisal, ultimately contributing to higher PTG levels. Conclusions: This study highlights the significant association between online social support and post-traumatic growth (PTG) during the COVID-19 pandemic, both directly and through the sequential mediating effects of gratitude and cognitive reappraisal. These findings underscore the importance of leveraging digital platforms to provide emotional and cognitive resources that support resilience and growth in times of large-scale adversity. Psychological interventions should prioritize cultivating gratitude and enhancing cognitive reappraisal skills as effective strategies to mitigate the negative psychological impacts of crises and facilitate PTG outcomes.

## 1. Introduction

The COVID-19 pandemic has profoundly impacted public health and psychological challenges worldwide (D. Zhang et al., 2021). However, alongside these challenges, individuals may also experience post-traumatic growth (PTG), referring to individuals’ positive transformations in areas such as self-perception, interpersonal relationships, and worldviews following traumatic events (Tedeschi & Calhoun, 1995).

Although PTG has been extensively studied in relation to common traumatic events, such as natural disasters and severe illnesses, research on PTG in the context of major public health crises is still limited. The COVID-19 pandemic, as a pervasive and long-lasting global crisis, has created a unique context for exploring how individuals achieve PTG under prolonged collective stress. Moreover, identifying specific factors and mechanisms that promote PTG in such circumstances is critical for designing interventions to enhance psychological resilience.

### 1.1. Online Social Support

The factors that promote PTG include the characteristics of traumatic events, individual factors, and environmental factors. Compared to the inherent characteristics of traumatic events and stable individual factors, environmental factors are more easily changed and intervened. A potential key factor in fostering PTG through adaptive coping mechanisms is online social support, a novel form of interpersonal interaction that has gained prominence during the pandemic due to restricted face-to-face communication.

The Theory of Crises and Personal Growth proposed by Schaefer and Moos (1992) suggests that environmental factors are key to whether individuals can achieve growth after crisis events. Among them, social support is the most important environmental factor, closely related to an individual’s post-traumatic psychological response (W. Liu et al., 2021). Adequate social support can provide a safe environment for trauma survivors to freely discuss their traumatic experiences and related emotions with others. Moreover, from the perspective of the Conservation of Resources Theory, social support is an important part of an individual’s social resources (Hobfoll & Shirom, 2001). Social support from others can also provide resources for post-traumatic individuals to compensate for the resources lost during trauma, thereby reducing the negative impact of traumatic events, helping them find benefits after trauma, and achieving post-traumatic growth. Therefore, social support can guide individuals to reconstruct their worldview after trauma, thereby enhancing PTG (X. Zhou et al., 2017c).

Previous research has mainly focused on offline support, that is, face-to-face support, to explore the relationship between social support and post-traumatic growth (Tedeschi & Calhoun, 2004; Helgeson, 2003). However, during the COVID-19 pandemic, traditional face-to-face support mechanisms have been constrained due to social distancing and isolation requirements. This unique context has highlighted the growing significance of online social support, which refers to the sense of belonging, understanding, and validation individuals gain through virtual communication (Liang & Wei, 2008).

Unlike offline support, which relies heavily on physical presence and non-verbal cues, online social support operates through text-based, asynchronous, and often anonymous interactions. These unique characteristics give rise to distinct psychological mechanisms. For instance, online platforms can provide a heightened sense of anonymity and reduced social anxiety, enabling individuals to express themselves more openly and seek support without fear of judgment (Barak et al., 2008). Additionally, the asynchronous nature of online communication allows individuals to process and respond to support at their own pace, which may enhance reflective thinking and cognitive coping strategies (Wright, 2016). Empirical studies have consistently demonstrated the positive effects of online social support. For example, it has been shown to maintain individual physical and mental health, improve wellbeing, alleviate mental health conditions, and buffer individuals’ loneliness (Gilmour et al., 2020; J. Fang et al., 2020). By fostering a sense of belonging within virtual spaces, online social support helps individuals adopt a more positive outlook and promotes social–psychological adaptation (Hofhuis et al., 2019; Q. Liu et al., 2016).

As the pandemic transitions into a phase of normalization, the limitations of traditional face-to-face support mechanisms have further accelerated the adoption of online social media platforms for interpersonal communication. In this regard, the pandemic has established online social support as a critical mechanism for maintaining interpersonal connections and accessing psychological resources. Importantly, the psychological mechanisms underlying online social support differ significantly from those of face-to-face interactions. For example, virtual groups and forums can foster a sense of community and shared experience, which is particularly beneficial for individuals who feel isolated or stigmatized in their offline environments (Nabi et al., 2013). Moreover, the ability to access diverse perspectives and experiences online can enhance cognitive reappraisal and emotional regulation, both of which are key processes in facilitating PTG (Tugade & Fredrickson, 2007). Yet, the specific role of online social support in facilitating PTG during major public health crises remains underexplored. This highlights the need to examine how online social support fosters PTG and the underlying psychological mechanisms. Thus, we propose Hypothesis 1 (H1): Online social support positively predicts PTG during the COVID-19 pandemic.

According to the social environment model of post-traumatic growth (PTG) (Helgeson & Lopez, 2010), individual growth is environmental in nature, and social support from others can directly promote positive evaluations of others, thereby further enhancing positive experiences of interpersonal relationships, emphasizing the direct role of social support in the realization of PTG. The effect of social support on PTG may also be influenced by individual factors. Joseph et al. (2012) complements the social environment model of PTG by viewing growth as the outcome of resolving challenging beliefs and recognizing that this process is facilitated by the combination of emotional states and cognitive activities of individuals in response to traumatic events. An individual’s emotional state plays a key role in the psychological adaptation process following trauma. In the context of COVID-19 pandemic control, gratitude is considered a typical positive emotion that is universally present in the growth process of trauma-exposed individuals. Concurrently, positive cognitive processes also help resolve the conflicts brought about by trauma, attributing positive meaning to negative events in a manner of personal growth. This cognitive strategy of directly confronting negative events and actively processing event information is termed cognitive reappraisal (Garnefski et al., 2001), which can further promote PTG. Based on the aforementioned theoretical frameworks, this study aims to delve into the intrinsic mechanisms between online social support and post-traumatic growth in the context of the pandemic. By analyzing the emotional states and cognitive activities of individuals facing pandemic challenges, as well as the role of online social support in this process, the study seeks to reveal the pathways through which online social support influences individual post-traumatic growth, providing theoretical support and practical guidance for future responses to similar public health emergencies.

### 1.2. Gratitude as a Mediator

While environmental factors like social support provide the foundation for coping, the individual’s emotional state plays an equally vital role in the process of achieving PTG. In particular, gratitude, which represents a state of appreciation and recognition for help received, has been identified as a key emotional response that promotes psychological resilience. During the pandemic, the numerous touching stories and heroic figures have sparked feelings of gratitude among people. Gratitude is an internal psychological state of appreciation that individuals experience after receiving help from others. It is an overt, immediate emotional experience that varies across different contexts (McCullough et al., 2002).

According to the broaden-and-build theory (Fredrickson, 2001), experiencing gratitude enhances positive emotions and broadens cognitive and behavioral flexibility, enabling individuals to identify positive aspects within negative events. Research suggests that gratitude is closely linked to social support; individuals who perceive high levels of support are more likely to feel grateful (He et al., 2015; Walther & Boyd, 2002; Yang et al., 2017). However, it is important to note that, according to the Find, Remind, and Bind Theory, gratitude does not automatically arise from social support unless the support is subjectively interpreted as meaningful assistance. This interpretation is critical, as it underscores the cognitive appraisal process through which perceived support is transformed into gratitude (Algoe, 2012). Furthermore, the Gratitude Amplification Theory posits that gratitude is a unique cognitive–emotional resource (Watkins, 2014). When individuals receive help, they tend to amplify the significance of the event, which can enhance their positive emotional experiences, protect emotional wellbeing, and promote self-acceptance. Gratitude is also significantly negatively correlated with various negative emotional traits, somatic symptoms, and aggressive behaviors (Ma & Hu, 2004), helping individuals to uncover and amplify the positive significance behind negative events, thus promoting personal growth. A substantial body of empirical research has demonstrated the close relationship between gratitude and individual adaptation and growth. For instance, Witvliet et al. (2019) found that gratitude can serve as a predictor of hope and happiness, fostering a sense of hopefulness and wellbeing.

Building on this, gratitude may serve as an important mechanism linking online social support and PTG. During the pandemic, gratitude has been shown to enhance wellbeing and foster personal growth, particularly among vulnerable groups, such as college students and trauma survivors (Xia et al., 2021; Geng & Qian, 2022; Wang & Wu, 2020). These findings suggest that gratitude not only provides emotional relief but also facilitates cognitive and emotional adjustments that promote positive psychological outcomes. Accordingly, we propose Hypothesis 2 (H2): Gratitude mediates the relationship between online social support and PTG.

### 1.3. Cognitive Reappraisal as a Mediator

In addition to its emotional impacts, social support also influences cognitive processes. Cognitive reappraisal, a strategy for reframing negative events (Gross, 2002), allows individuals to reinterpret trauma in a way that highlights personal growth opportunities. When individuals encounter negative events, they can employ cognitive reappraisal strategies to form new understandings of the event by changing their past cognitions. Social support can satisfy individuals’ relational needs, help them gain more social resources, and significantly influence cognitive strategies. Adolescents with strong social support are more inclined to adopt positive cognitive strategies, such as cognitive reappraisal; in contrast, those lacking social support are more likely to frequently use negative cognitive strategies, such as self-blame and suppression (Li et al., 2021). The routine application of cognitive reappraisal brings a positive “spillover effect”, enhancing emotional stability and contributing to the maintenance of mental and physical health (Brockman et al., 2016). When facing trauma, cognitive reappraisal can prompt individuals to actively reconsider the entire event, form a new life narrative, and ultimately foster the emergence of post-traumatic growth (PTG) (Tian et al., 2018). The Social Environment Model of PTG (Helgeson & Lopez, 2010) emphasizes that supportive environments promote cognitive reappraisal by providing the psychological safety needed to process traumatic experiences constructively. Particularly during trauma, cognitive reappraisal enables individuals to create a coherent life narrative, transforming their perspectives on adverse events and facilitating PTG (L. Zhang et al., 2018). Given the unique capacity of online social support to provide a safe and supportive environment, it is reasonable to posit that cognitive reappraisal plays a mediating role in the relationship between social support and PTG. Hence, we propose Hypothesis 3 (H3): Cognitive reappraisal mediates the relationship between online social support and PTG.

### 1.4. The Chain Mediation of Gratitude and Cognitive Reappraisal

Finally, from the above theoretical analysis, it can be inferred that gratitude and cognitive reappraisal may interact to form a chain mediation mechanism linking online social support to PTG. Is there a relationship between gratitude and cognitive reappraisal? The affective-cognitive processing model of PTG does not clarify the relationship between gratitude and cognitive reappraisal. This study intends to further explore the relationship between gratitude and cognitive reappraisal. On the one hand, the affective-cognitive processing model of PTG (Joseph et al., 2012) believes that a specific emotional state will lead to the action of the cognitive assessment mechanism, resulting in positive or negative cognitive activities. Individuals’ emotions after traumatic events lead to the emergence of corresponding cognitive coping strategies. On the other hand, the extended construction theory of gratitude points out (Fredrickson, 2004) that gratitude will make individuals have a positive tendency to view and know the events around them, expanding the cognitive range of individuals (Fredrickson & Branigan, 2005). Gratitude contributes to the internalization of positive experiences, which can enhance psychological resources and resilience. By reinforcing a focus on beneficial aspects of situations, gratitude may support positive cognitive appraisals and adaptive coping strategies (Hanson et al., 2023). Additionally, empirical evidence further supports this causal relationship. For example, an experimental daily diary study demonstrated that gratitude interventions significantly increase the use of cognitive reappraisal strategies, which in turn enhance wellbeing outcomes (Hartanto et al., 2023). Similarly, Bryan et al. (2018) found that gratitude alleviates the negative impact that emotional expression conflicts on depression by promoting cognitive reappraisal. Moreover, research indicated that a grateful mindset can facilitate the reconstruction of one’s self-identity, leading to a positive transformation of self-image (Froh et al., 2009). These findings provide robust evidence that gratitude can directly influence cognitive reappraisal processes. Therefore, the supportive environment provided by online social support is conducive to generating positive emotions (such as gratitude), further promotes the cognitive reappraisal of traumatic events, and finally, encourages the achievement of PTG. Therefore, this study proposes Hypothesis 4 (H4): Online social support can predict PTG through the chain mediation of gratitude and cognitive reappraisal.

### 1.5. Research Objectives

**Figure 1:**
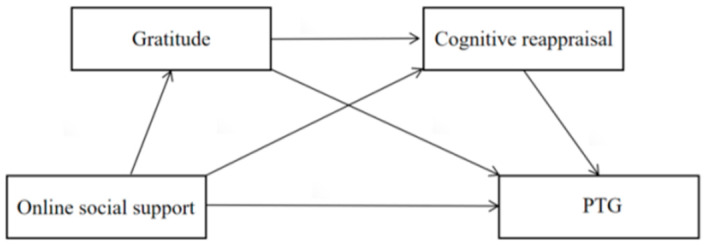
Hypothetical model of the serial mediation effect.

In summary, this study integrates theoretical perspectives to examine how online social support influences PTG during the COVID-19 pandemic and the mediating roles of gratitude and cognitive reappraisal. Based on the social environment model of PTG and the cognitive-affective model of PTG, this study proposes a serial mediating model (see Figure 1). It explores the impact of online social support on individual post-traumatic growth and the role of gratitude and cognitive reappraisal therein. It seeks to deepen our understanding of the psychological mechanisms underlying PTG and provide practical guidance for enhancing mental health interventions during public health crises.

## 2. Materials and Methods

### 2.1. Study Design

Through the star network questionnaire survey, an anonymous online survey was conducted from 14 March to 31 March 2022 during the acute phase in which the frequency of local outbreaks increased significantly, and the affected area was large^1^. The questionnaire star platform was used to design the questionnaire, and the questionnaire was completed online through WeChat and QQ platforms. The purpose, content, selection method, and matters needing attention are stated in the instructions. To improve the questionnaire answer rate, the answers are set to have a 50% chance of getting cash rewards. To ensure the integrity and effectiveness of the questionnaire, all items are set as required questions, and one account, one device, and one IP address can only be filled in once.

### 2.2. Participants and Data Collection

In this study, we recruited college students who responded to our questionnaires between 14 March and 31 March 2022. A total of 607 questionnaires were distributed, and 545 valid questionnaires were obtained with an effective recovery rate of 89.79%. There were 159 males (29.17%) and 386 females (70.83%). Although our sample shows a gender imbalance (70.83% female vs. 29.17% male), this exploratory study primarily focuses on examining the relationships between variables rather than gender differences. To control for potential gender effects, we included gender as a covariate in our analyses. The home address was 256 (46.97%) in rural areas and 289 (53.03%) in urban areas. There were 116 close contacts (21.28%) and 48 confirmed cases (8.81%) in the family/community.

### 2.3. Ethical Approval

Ethical approval was obtained from the Department and Institute of Psychology, Ningbo University. We obtained written informed consent from participants, and we ensured their privacy and confidentiality. Participants were then offered debriefing sessions with the main investigator where needed, and those with psychological disturbances were advised counselling sessions in the hospital’s psychiatric clinic.

### 2.4. Measures

The College Students’ Online Social Support Testing Questionnaire compiled by Liang and Wei (2008) was adapted, according to the pandemic situation and the actual online social support received by the public in China, with a total of 23 items. It includes four dimensions: companion support, emotional support, instrumental support, and information support. A 5-point scale was used, where 1 means “never” and 5 means “always”. Higher scores indicate more online social support. In this study, the internal consistency coefficient of the scale was 0.94, and the internal consistency coefficient of each dimension ranged from 0.79 to 0.92.

The Post-Traumatic Growth Inventory (PTGI), revised by Y. Zhang et al. (2013) and compiled by Tedeschi and Calhoun (1996), has a total of 21 items. It includes the following five dimensions: interpersonal relationship, new possibilities, personal power, appreciation of life, and spiritual change. A 5-point scale was used, where 1 means “not at all” and 5 means “to a very large extent”. Higher scores indicate higher levels of PTG. In this study, the internal consistency coefficient of the scale was 0.94, and the internal consistency coefficient of each dimension ranged from 0.63 to 0.84.

The Cognitive Reappraisal Scale, part of the Cognitive Emotion Regulation Questionnaire (CERQ) compiled by Zhu et al. (2007), was used, with a total of four items. A 5-point scale was used, where 1 indicated “not at all” and 5 indicated “to a very large extent”. Higher scores indicate higher levels of cognitive reappraisal. In this study, the internal consistency coefficient of the scale was 0.86.

The Gratitude Adjective Checklist (GAC), a state gratitude measurement tool compiled by McCullough et al. (2002), is used to reflect individuals’ immediate gratitude emotional experience to assess three emotional adjectives (“thankful”, “grateful”, and “appreciative”) and their matching degree. A 5-point scale is used, where 1 means “not at all” and 5 means “to a very large extent”. Higher scores indicate higher levels of PTG. In this study, the internal consistency coefficient of the scale was 0.92.

### 2.5. Statistical Analysis

SPSS 21.0 was used to analyze the data in this study. Before testing the hypothesis, we eliminated invalid samples according to the reverse scoring items and the repetition rate of answers. In addition, there were no data missing in this study. Descriptive analyses were performed on all variables, and Pearson correlation analyses were conducted to test the correlations among the main variables. Next, Model 6 in PROCESS plug-in was used to study the chain mediation model, and the Bootstrap method was used to test the significance of regression coefficients in the parameter test. According to the results of the correlation analysis, gender, pandemic exposure, and home address were controlled as covariates in the analyses.

As for the common method biases test, in issuing questionnaires, this study controlled the influence of common method deviation using anonymous questionnaires, the separate typesetting of questionnaires, and the reverse scoring of some questions in advance (H. Zhou & Long, 2004). After data collection, the Harman single factor test was used to conduct a statistical test after common method deviation (Podsakoff et al., 2003). In addition, the results show that the eigenvalues of seven factors obtained by rotation are greater than 1, and the variation of the explanatory rate of the first factor is 21.59%, which is lower than the critical value of 40%, indicating that there is no obvious common method bias in this study.

## 3. Results

### 3.1. Descriptive Statistics and Correlation Analysis of Variables

The average PTG score of 545 participants was (3.59 ± 0.60), and the average scores of PTG and each dimension of the participants were above 3.40 (the score range was 1–5). The level of individual PTG was high during the pandemic. Among them, interpersonal relationships scored (3.54 ± 0.61), new possibilities (3.49 ± 0.70), personal power (3.61 ± 0.66), spiritual change (3.61 ± 0.72), and appreciation of life (3.82 ± 0.72).

The descriptive statistics and correlation analysis results of each variable in this study are shown in Table 1. Among them, there was a significant positive correlation between pandemic exposure and online social support and a significant negative correlation between home address and PTG. Therefore, pandemic exposure and home address were controlled as covariates in subsequent analyses (Zhen & Zhou, 2020). In addition, there was a significant positive correlation between online social support, gratitude, cognitive reappraisal, and PTG.

### 3.2. Chain Mediation Model Test

Since the results of correlation analysis meet the statistical requirements for further mediating effects of gratitude and cognitive reappraisal (Wen & Ye, 2014), the PROCESS was then used to perform the bootstrap-based mediation effect test.

The results of regression analysis showed that, first, online social support significantly positively predicted PTG (*p* < 0.001), significantly positively predicted gratitude (*p* < 0.001), and significantly positively predicted cognitive reassessment (*p* < 0.001); second, gratitude significantly positively predicted cognitive reappraisal (*p* < 0.001) and significantly positively predicted PTG (*p* < 0.001); finally, cognitive reappraisal significantly positively predicted PTG (*p* < 0.001).

**Figure 2:**
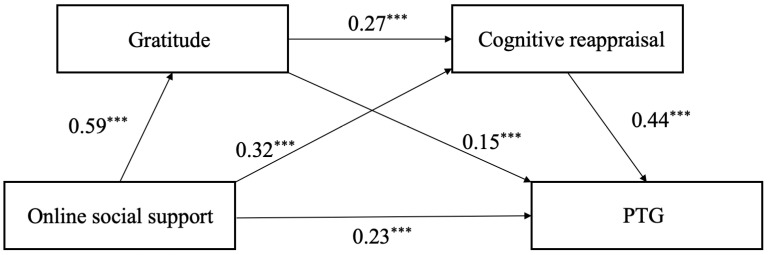
Chain mediating effects of gratitude and cognitive reappraisal online social support on PTG. Note: *** *p* < 0.001.

The analysis and test of the mediating effect results show that the 95% confidence intervals of the three paths do not contain 0, indicating that the three indirect effects have all reached a significant level, and the chain mediation model is established (see Figure 2). The path coefficient results are shown in Table 2. Specifically, the indirect effect of the path with gratitude as the mediating variable was 0.09 (95%CI = [0.05, 0.12]). The indirect path effect of cognitive reappraisal was 0.14 (95%CI = [0.09, 0.19]). The indirect path effect of gratitude and cognitive reassessment as mediating variables was 0.07 (95%CI = [0.04, 0.10]).

In general, this study found that, in addition to directly predicting PTG, online social support could also predict PTG through three pathways: gratitude, cognitive reappraisal, and the chain mediation of gratitude and cognitive reappraisal. Among all the pathways, the direct pathway of online social support had the largest effect size, accounting for 43.40% of the total effect. Online social support could directly predict the generation of PTG. Among the indirect pathways, the independent mediation pathway of cognitive reappraisal was the largest, accounting for 26.42% of the total effect. The independent mediating path of gratitude was second, accounting for 16.98% of the total effect. The chain mediators of gratitude and cognitive reappraisal had the smallest effect size, accounting for 13.21% of the total effect. It can be seen that cognitive reappraisal plays the largest role between online social support and PTG, which indicates that online social support is positively associated with individual growth, mainly through encouraging individuals to adopt positive cognitive coping strategies.

## 4. Discussion

This study examined the relationship between online social support, gratitude, cognitive reappraisal, and post-traumatic growth (PTG) during the COVID-19 pandemic, a sudden public health event. It was found that online social support can directly and positively predict PTG, as well as positively predict PTG through the mediation of gratitude and cognitive reappraisal separately, and also positively predict PTG through the serial mediation of gratitude and cognitive reappraisal. These significant findings not only emphasize the crucial role of online social support in facilitating individual post-traumatic growth but also reveal the mediating mechanisms of gratitude and cognitive reappraisal, offering a new perspective for psychological health interventions, especially in the context of current and future potential public health emergencies.

### 4.1. Online Social Support Positively Predicts PTG

This study explored the impact of online social support on post-traumatic growth (PTG) in the context of the COVID-19 pandemic and confirmed Hypothesis H1, which posits that online social support positively predicts PTG. This finding is consistent with previous research, emphasizing the key role of social support in facilitating positive changes in individuals after trauma (Schaefer & Moos, 1992), and further supports the social environment model and cognitive-affective model of PTG (Calhoun & Tedeschi, 2006). The model highlights that online social support provides individuals with a safe atmospheric environment and instrumental support to cope with trauma, factors that are positively associated with the realization of PTG. From the perspective of the crisis–growth theory (Schaefer & Moos, 1992), the online social support system during the pandemic creates a secure atmosphere for individuals, helping to alleviate stress and negative emotions caused by the pandemic, thereby supporting PTG. According to the survey, approximately 95% of people used at least one form of online media (such as Weibo, WeChat) for communication during the pandemic. Online support plays a role through various avenues: individuals can gain companionship support through online chatting, watching movies together, and playing games; emotional support by expressing emotions and feelings; informational support by obtaining advice, guidance, and situational assessments regarding coping with COVID-19; or instrumental support by receiving financial or material aid (such as masks and disinfectants) (Zhong et al., 2021). In the COVID-19 pandemic control, multiple psychological support hotlines were launched nationwide, governments and universities actively explored online teaching mechanisms, and relevant departments conducted online mental health education for the public, providing strong online social support.

The survey revealed that individuals’ PTG after the pandemic was at a moderate level, with an average score of 3.59 ± 0.60. Most participants felt that they had experienced growth during the pandemic, leading to new contemplations about life, living, and existence. Compared to the PTG scores after the Wenchuan and Ya’an earthquakes (X. Zhou et al., 2014, 2017b, 2019), the level of PTG triggered by public health events was higher. This may be due to the more enduring and widespread impact of public health events. In contrast to natural disasters, like earthquakes, public health events often have profound effects on multiple aspects of society, economy, environment, and individual life. This enduring and widespread impact may prompt individuals to adopt a more proactive attitude during the adaptation and adjustment process, thereby facilitating post-traumatic growth.

### 4.2. Mediating Effects of Gratitude and Cognitive Reappraisal on Online Social Support and PTG

This study delved into the mediating mechanisms between online social support and post-traumatic growth (PTG), finding that both gratitude and cognitive reappraisal played mediating roles in the process, providing a new perspective for understanding the mechanism by which online social support contributes to growth after trauma.

Firstly, gratitude, as an emotion that focuses on and appreciates the positive aspects of life, plays a crucial role in individuals’ psychosocial adaptation (Tsang et al., 2014). In this study, online social support facilitated the development of PTG by enhancing individuals’ feelings of gratitude, which not only confirmed Hypothesis H2 but also aligned with previous research findings showing a significant association between online social support and positive emotions (Tian et al., 2017). According to the broaden-and-build theory of positive emotions (Fredrickson, 2001), gratitude can broaden individuals’ thinking and behavioral patterns, helping to establish lasting physical, psychological, and social resources, improve the quality of interpersonal relationships, and enhance positive evaluations of oneself, others, and the world, thereby being positively associated with the formation of PTG. In fact, during the pandemic, many touching stories emerged from all sectors of society, with brave individuals going against the flow, people sharing weal and woe, and nationwide mutual support, providing abundant online social support. These deeply moving stories were widely reported and praised, greatly fostering a sense of gratitude across society.

Secondly, as an effective emotional regulation strategy, cognitive reappraisal helps individuals to positively re-evaluate their traumatic experiences, thereby being positively associated with PTG, which supports Hypothesis H3. This finding further adds further support to the social cognitive processing theory (Lepore & Greenberg, 2002). The theory suggests that the material and emotional supportive environment provided by online social support is associated with individuals’ ability to cognitively re-evaluate the pandemic and its negative consequences, deepening positive thinking and meaning integration about the pandemic, mitigating the negative impact of this public health event, and being associated with positive transformation after trauma. Additionally, existing research has confirmed that cognitive reappraisal plays a significant mediating role between social support and PTG (Roni et al., 2020; X. Zhou et al., 2017a). As the social environment model of PTG states, compared to traditional offline social support, online social support is more likely to be associated with transforming the cognitive patterns of individuals after trauma, enabling a deep exploration of the meaning of traumatic events and their outcomes (Cao et al., 2014). By more frequently employing cognitive reappraisal strategies, people can re-envision the significance the pandemic has given to them, enhance trust in their own abilities, and promote group unity and mutual support, strengthening interpersonal connections. Moreover, individuals can also rethink their lives and existence after the pandemic’s impact, exploring new possibilities. All these re-recognitions and evaluations of traumatic events, the self, and others contribute to individuals achieving PTG (Tian et al., 2018).

Furthermore, more importantly, this study found that online social support can indirectly predict PTG through the serial mediation of gratitude and cognitive reappraisal, indicating that, under the influence of online social support, the enhancement of gratitude may stimulate individuals’ cognitive reappraisal processes, thereby jointly supporting the realization of PTG. This finding confirms Hypothesis H4. According to the cognitive-affective model of PTG (Joseph et al., 2012), emotional states affect the choice and application of cognitive strategies, with positive emotions such as gratitude prompting the use of cognitive reappraisal strategies, leading to positive transformation after trauma. At the same time, under the influence of online social support, individuals after trauma may experience intense feelings of gratitude, which help expand their attention, cognition, and action range (Fredrickson, 2004), enabling them to go beyond the negative aspects of the pandemic, thus aiding in liberating themselves from the negative impacts of the pandemic, enhancing positive cognition of the real world, and adopting more effective coping strategies to deal with issues during the post-trauma adaptation process, thereby achieving adaptation and growth in the real world. Therefore, the serial mediation of gratitude and cognitive reappraisal is also an important mechanism by which online social support affects PTG. These findings extend prior research by elucidating the interplay between emotional and cognitive mechanisms, demonstrating how online social support enables this dynamic process.

It is essential to recognize that the cross-sectional nature of our data imposes certain speculative limitations regarding the causal pathways we have proposed. While we have posited a direction from online social support to gratitude to cognitive reappraisal and, ultimately, PTG, it is equally plausible that the relationship works in reverse. Specifically, gratitude may enhance the perception of social support, leading individuals to feel more supported (Lin, 2016).

Several studies have indicated that individuals exhibiting higher levels of gratitude are more likely to perceive and receive greater social support from a range of sources, including family, friends, and even strangers (Wood et al., 2008; Froh et al., 2009). Furthermore, the process of cognitive reappraisal may also contribute to feelings of gratitude. When individuals actively reframe their thoughts about various situations or their interactions with others, they create new cognitive frameworks that can adjust their emotional responses. This newly formed cognitive perspective can bolster an individual’s sense of gratitude and appreciation towards others. Empirical evidence demonstrates the influence of cognitive reappraisal on enhancing state gratitude through multiple experimental approaches, including motivational reappraisal, cost reappraisal, and value reappraisal, which collectively illustrate its impact from various angles (P. Zhang & Zhang, 2016).

Given the possible reciprocal relationships among these constructs, further exploration through longitudinal studies is warranted to more thoroughly investigate the dynamics between online social support, gratitude, cognitive reappraisal, and PTG.

### 4.3. Research Significance

Overall, this study found that online social support can directly and positively predict post-traumatic growth (PTG), as well as positively predict PTG through the mediation of gratitude and cognitive reappraisal separately, and also positively predict PTG through the serial mediation of gratitude and cognitive reappraisal. Theoretically, this research integrates the social environment model of PTG and the cognitive-affective model of PTG, providing empirical evidence for the prediction of individual post-traumatic growth by online social support, deepening the understanding of social support and post-traumatic growth theories, further revealing the mechanisms of individual post-traumatic growth under pandemic conditions, and offering theoretical guidance for psychosocial rehabilitation efforts during public health emergencies.

On the practical level, the findings of this study provide guidance for mental health interventions and public policy. First, they emphasize the potential of online platforms to provide effective social support during crises. Policymakers and practitioners should consider developing virtual support networks that prioritize emotional validation and resource exchange. For example, interventions such as online gratitude journal, community-building platforms, and forums designed to encourage cognitive reappraisal could significantly enhance individuals’ resilience and growth. Second, the study highlights the value of integrating gratitude and cognitive reappraisal training into psychological interventions. Online programs that teach individuals to recognize and express appreciation, as well as reframe negative experiences, could amplify the benefits of virtual social support and maximize PTG outcomes.

### 4.4. Limitations and Future Directions

Despite its contributions, this study has several limitations that require further exploration. First, the cross-sectional design limits the ability to infer causality. In fact, existing research has demonstrated that individuals may develop adaptive responses to social support, and over time, their sensitivity to the perception of social support may decrease (Prati & Pietrantoni, 2009; Butler et al., 2005). Furthermore, the continued presence of social support long after a traumatic experience might cause individuals to lose the proactivity in recognizing and making meaning in the post-trauma world (Wu et al., 2016). Future longitudinal studies are needed to establish the temporal dynamics of the relationships observed in this study. Second, a notable limitation of this study is the uneven gender distribution in our sample, which may affect the generalizability of our findings. While we controlled for gender effects statistically, future research should address this limitation through more balanced sampling approaches. Future studies should employ gender-stratified sampling methods with predetermined quotas to achieve a more balanced gender representation. Additionally, conducting separate analyses for male and female participants with adequate sample sizes would allow for meaningful gender comparison in PTG development. The study also did not account for other stressors or psychological issues that participants may have experienced after the outbreak. Subsequent research should consider incorporating these factors into the analysis to more fully understand the impact of online social support on PTG. Furthermore, the reliance on self-reported data introduces the potential for social desirability bias and recall bias. Future research could employ objective measurement methods or collect data from various sources to mitigate these biases and enhance the validity of findings. Finally, considering the influence of cultural factors on gratitude and social support, we suggest that future research explore how cultural differences shape these psychological mechanisms. Investigating these cultural variations will provide deeper insights into the mechanisms of post-traumatic growth across different cultural contexts.

## 5. Conclusions

Based on the social environment model of PTG and the cognitive-affective model of PTG, this study explored the relationship between online social support and PTG and its underlying mechanism. The findings revealed that online social support could positively predict the development of PTG. The role of online social support, gratitude, and cognitive reappraisal, as positive emotional and cognitive processes, can further promote positive changes in post-traumatic individuals. In addition, online social support can also indirectly affect PTG through the chain mediation of gratitude and cognitive reappraisal. These results suggest that psychological intervention under the pandemic can focus on the awakening of gratitude and the use of cognitive reappraisal strategies to reduce the negative impact of the pandemic on individuals and promote the realization of PTG.

## Figures and Tables

**Table 1:** Mean values, standard deviations, and correlation coefficients of each variable (*n* = 545).

Variables	*M*	*SD*	1	2	3	4	5	6	7
1. Gender ^a^	0.71	0.46	–						
2. Home address ^b^	0.53	0.50	0.01	–					
3. Isolating experience ^c^	0.30	0.46	−0.01	−0.06	–				
4. COVID-19 exposure	1.70	0.60	0.17 ***	−0.03	0.05	–			
5. Online social support	3.33	0.69	−0.07	−0.01	−0.02	−0.08	–		
6. Gratitude	3.76	0.90	0.04	−0.07	−0.03	0.01	0.45 ***	–	
7. Cognitive reappraisal	3.77	0.67	0.06	−0.08	−0.03	0.01	0.50 ***	0.52 ***	–
8. PTG	3.59	0.60	0.01	−0.10 *	0.05	−0.03	0.60 ***	0.59 ***	0.73 ***

Note: “^a^” is a dummy variable, 0 = male, 1 = female; “^b^” is a dummy variable, 0 = urban, 1 = rural; “^c^” is the dummy variable, 0 = home isolation/dormitory isolation, 1 = non-home isolation/centralized isolation; * *p* < 0.05, *** *p* < 0.001, same as below.

**Table 2:** Path coefficient analysis of mediation model.

Mediation Path	Mediating Effect Value	Standard Error	95% Confidence Interval
*X*→*M*_1_→*Y*	0.09	0.02	[0.05, 0.12]
*X*→*M*_2_→*Y*	0.14	0.02	[0.09, 0.19]
*X*→*M*_1_→*M*_2_→*Y*	0.07	0.01	[0.04, 0.10]
Total indirect effect	0.30	0.03	[0.24, 0.36]
Indirect effect	0.23	0.03	[0.18, 0.28]
Total effect	0.53	0.03	[0.47, 0.59]

Note: *X* = online social support, *M*_1_ = gratitude, *M*_2_ = cognitive reappraisal, *Y* = post-traumatic growth (PTG).

## Data Availability

The raw data supporting the conclusions of this article will be made available by the authors on request.

## Note

1 http://www.gov.cn/xinwen/gwylflkjz186/index.htm (accessed on 25 March 2022).

## References

[B1-behavsci-15-00335] Algoe S. B. (2012). Find, remind, and bind: The functions of gratitude in everyday relationships. Social and Personality Psychology Compass.

[B2-behavsci-15-00335] Barak A., Boniel-Nissim M., Suler J. (2008). Fostering empowerment in online support groups. Computers in Human Behavior.

[B3-behavsci-15-00335] Brockman R., Ciarrochi J., Parker P., Kashdan T. (2016). Emotion regulation strategies in daily life: Mindfulness, cognitive reappraisal and emotion suppression. Cognitive Behaviour Therapy.

[B4-behavsci-15-00335] Bryan J. L., Young C. M., Lucas S., Quist M. C. (2018). Should I say thank you? Gratitude encourages cognitive reappraisal and buffers the negative impact of ambivalence over emotional expression on depression. Personality and Individual Differences.

[B5-behavsci-15-00335] Butler L. D., Blasey C. M., Garlan R. W., McCaslin S. E., Azarow J., Chen X.-H., Desjardins J. C., DiMiceli S., Seagraves D. A., Hastings T. A., Kraemer H. C., Spiegel D. (2005). Posttraumatic growth following the terrorist attacks of September 11, 2001: Cognitive, coping, and trauma symptom predictors in an internet convenience sample. Traumatology.

[B6-behavsci-15-00335] Calhoun L. G., Tedeschi R. G., Calhoun L. G., Tedeschi R. G. (2006). The foundations of posttraumatic growth: An expanded framework. Handbook of posttraumatic growth.

[B7-behavsci-15-00335] Cao C., Wang M., Zhang W., Ji L., Chen L., Chen X. (2014). The interactive effect of COMT gene rs6267 polymorphism and maternal parenting behavior on adolescents’ physical and relational aggression. Acta Psychologica Sinica.

[B8-behavsci-15-00335] Fang J., Wang X. C., Wen Z. L., Huang J. Y. (2020). Cybervictimization and loneliness among Chinese college students: A moderated mediation model of rumination and online social support. Children and Youth Services Review.

[B9-behavsci-15-00335] Fredrickson B. L. (2001). The role of positive emotions in positive psychology: The broaden-and-build theory of positive emotions. American Psychologist.

[B10-behavsci-15-00335] Fredrickson B. L., Emmons R. A., McCullough M. E. (2004). Gratitude, like other positive emotions, broadens and builds. The psychology of gratitude.

[B11-behavsci-15-00335] Fredrickson B. L., Branigan C. (2005). Positive emotions broaden the scope of attention and thought-action repertoires. Cognition and Emotion.

[B12-behavsci-15-00335] Froh J. J., Kashdan T. B., Ozimkowski K. M., Miller N. (2009). Who benefits the most from a gratitude intervention in children and adolescents? Examining positive affect as a moderator. The Journal of Positive Psychology.

[B13-behavsci-15-00335] Garnefski N., Kraaij V., Spinhoven P. (2001). Negative life events, cognitive emotion regulation and emotional problems. Personality and Individual Differences.

[B14-behavsci-15-00335] Geng X., Qian Y. (2022). The mediating effect of gratitude on psychological resilience and posttraumatic growth among patients undergoing radical lung cancer surgery. General Nursing.

[B15-behavsci-15-00335] Gilmour J., Machin T., Brownlow C., Jeffries C. (2020). Facebook-based social support and health: A systematic review. Psychology of Popular Media.

[B16-behavsci-15-00335] Gross J. J. (2002). Emotion regulation: Affective, cognitive, and social consequences. Psychophysiology.

[B17-behavsci-15-00335] Hanson R., Shapiro S., Hutton-Thamm E., Hagerty M. R., Sullivan K. P. (2023). Learning to learn from positive experiences. The Journal of Positive Psychology.

[B18-behavsci-15-00335] Hartanto A., Kaur M., Kasturiratna K. S., Quek F. Y., Majeed N. M. (2023). A critical examination of the effectiveness of gratitude intervention on well-Being Outcomes: A within-person experimental daily diary approach. The Journal of Positive Psychology.

[B19-behavsci-15-00335] He A., Hui Q., Liu H. (2015). The relationship between social support and loneliness among college students: The mediating role of gratitude. Chinese Journal of Clinical Psychology.

[B20-behavsci-15-00335] Helgeson V. S. (2003). Social support and quality of life. Quality of Life Research.

[B21-behavsci-15-00335] Helgeson V. S., Lopez L., John W. R., Alex J. Z., Hall. J. S. (2010). Social support and growth following adversity. Handbook of adult resilience.

[B22-behavsci-15-00335] Hobfoll S. E., Shirom A., Golembiewski R. T. (2001). Conservation of resources theory: Applications to stress and management in the workplace. Handbook of organizational behavior.

[B23-behavsci-15-00335] Hofhuis J., Hanke K., Rutten T. (2019). Social network sites and acculturation of international sojourners in the Netherlands: The mediating role of psychological alienation and online social support. International Journal of Intercultural Relations.

[B24-behavsci-15-00335] Joseph S., Murphy D., Regel S. (2012). An affective-cognitive processing model of post-traumatic growth. Clinical Psychology and Psychotherapy.

[B25-behavsci-15-00335] Lepore S. J., Greenberg M. A. (2002). Mending broken hearts: Effects of expressive writing on mood, cognitive processing, social adjustment and health following a relationship breakup. Psychology and Health.

[B26-behavsci-15-00335] Li J., Yao M., Liu H. (2021). From social support to adolescents’ subjective well-being: The mediating role of emotion regulation and prosocial behavior and gender difference. Child Indicators Research.

[B27-behavsci-15-00335] Liang X., Wei L. (2008). Preliminary exploration of online social support among college students. Psychological Science.

[B28-behavsci-15-00335] Lin C. C. (2016). The roles of social support and coping style in the relationship between gratitude and well-being. Personality and Individual Differences.

[B29-behavsci-15-00335] Liu Q., Xu Q., Liu H., Liu Q. (2016). The relationship between online social support and online altruistic behavior among college students: A moderated mediation model. Psychological Development and Education.

[B30-behavsci-15-00335] Liu W., Li L., Song J. (2021). A meta-analysis of the relationship between social support, psychological resilience, and posttraumatic growth. Chinese Journal of Health Psychology.

[B31-behavsci-15-00335] Ma Y., Hu Y. (2004). Preliminary development of a gratitude scale for college students. Chinese Journal of Health Psychology.

[B32-behavsci-15-00335] McCullough M. E., Emmons R. A., Tsang J. (2002). The grateful disposition: A conceptual and empirical topography. Journal of Personality and Social Psychology.

[B33-behavsci-15-00335] Nabi R. L., Prestin A., So J. (2013). Facebook friends with (health) benefits?: Exploring the palliative effects of social network sites compared to interpersonal networks. Cyberpsychology, Behavior, and Social Networking.

[B34-behavsci-15-00335] Podsakoff P. M., MacKenzie S. B., Lee J. Y., Podsakoff N. P. (2003). Common method biases in behavioral research: A critical review of the literature and recommended remedies. Journal of Applied Psychology.

[B35-behavsci-15-00335] Prati G., Pietrantoni L. (2009). Optimism, social support, and coping strategies as factors contributing to posttraumatic growth: A meta-analysis. Journal of Loss and Trauma.

[B36-behavsci-15-00335] Roni L. R., Sivan G. L., Malka M. (2020). Social participation and posttraumatic growth: The serial mediation of hope, social support, and reappraisal. Journal of Community Psychology.

[B37-behavsci-15-00335] Schaefer J. A., Moos R. H., Carpenter B. N. (1992). Life crises and personal growth. Personal coping: Theory, research, and application.

[B38-behavsci-15-00335] Tedeschi R. G., Calhoun L. G. (1995). Trauma and transformation: Growing in the aftermath of suffering.

[B39-behavsci-15-00335] Tedeschi R. G., Calhoun L. G. (1996). The posttraumatic growth inventory: Measuring the positive legacy of trauma. Journal of Traumatic Stress.

[B40-behavsci-15-00335] Tedeschi R. G., Calhoun L. G. (2004). Posttraumatic growth: Conceptual foundations and empirical evidence. Psychological Inquiry.

[B41-behavsci-15-00335] Tian Y., Hong Y., Niu G., Fan C. (2017). The impact of online social support on life satisfaction among vocational students: The mediating roles of just-world belief and gratitude. Studies of Psychology and Behavior.

[B42-behavsci-15-00335] Tian Y., Wu X., Wang W., Zhou X. (2018). The impact of adolescent attachment on posttraumatic stress disorder and posttraumatic growth: The mediating roles of cognitive reappraisal and expressive suppression. Psychological Development and Education.

[B43-behavsci-15-00335] Tsang J. A., Carpenter T. P., Roberts J. A., Frisch M. B., Carlisle R. D. (2014). Why are materialists less happy? The role of gratitude and need satisfaction in the relationship between materialism and life satisfaction. Personality and Individual Differences.

[B44-behavsci-15-00335] Tugade M. M., Fredrickson B. L. (2007). Regulation of positive emotions: Emotion regulation strategies that promote resilience. Journal of Happiness Studies.

[B45-behavsci-15-00335] Walther J. B., Boyd S., Lin C. A., Atkin D. J. (2002). Attraction to computer-mediated social support. Communication technology an society: Audience adoption and uses.

[B46-behavsci-15-00335] Wang W., Wu X. (2020). The impact of empathy on prosocial behavior among post-disaster adolescents: The mediating roles of gratitude, social support, and posttraumatic growth. Acta Psychologica Sinica.

[B47-behavsci-15-00335] Watkins P. C., Watkins P. C. (2014). Does social well-being?. Gratitude and the good life gratitude enhance.

[B48-behavsci-15-00335] Wen Z., Ye B. (2014). Analysis of mediation effects: Development of methods and models. Advances in Psychological Science.

[B49-behavsci-15-00335] Witvliet C., Richie F., Luna L., Tongeren D. (2019). Gratitude predicts hope and happiness: A two–study assessment of traitsand states. Journal of Positive Psychology.

[B50-behavsci-15-00335] Wood A. M., Maltby J., Gillett R., Linley P. A., Joseph S. (2008). The role of gratitude in the development of social support, stress, and depression: Two longitudinal studies. Journal of Research in Personality.

[B51-behavsci-15-00335] Wright K. B. (2016). Communication in health-related online social support groups/communities: A review of research on predictors of participation, applications of social support theory, and health outcomes. Review of Communication Research.

[B52-behavsci-15-00335] Wu X., Zhou X., Chen J., Zeng M., Tian Y. (2016). The relationship between social support, rumination, and posttraumatic growth: A longitudinal study based on adolescents after the Wenchuan earthquake. Psychological Science.

[B53-behavsci-15-00335] Xia Y., He W., Qian J. (2021). The impact of gratitude and social support on posttraumatic growth among college students after the pandemic. Journal of Southwest University (Natural Science Edition).

[B54-behavsci-15-00335] Yang X., Liu Q., Zhou Z. (2017). The impact of online social support on online altruistic behavior among college students: The roles of gratitude and social identity. Psychological Development and Education.

[B55-behavsci-15-00335] Zhang D., Wu X., Tian Y., Zeng M. (2021). The impact of adolescents’ emotional regulation difficulties on posttraumatic stress disorder symptoms: The mediating roles of intrusive rumination and state hope. Chinese Journal of Clinical Psychology.

[B56-behavsci-15-00335] Zhang L., Cai D., Zhao J., Xu Z., Xu N. (2018). The relationship between social support, depression, and problem behavior among adolescents: A moderated mediation model. Journal of Educational Biology.

[B57-behavsci-15-00335] Zhang P., Zhang M. (2016). The effect of cognitive reappraisal intervention on state gratitude in college students. Chinese Journal of Clinical Psychology.

[B58-behavsci-15-00335] Zhang Y., Wang J., Zhou Y., Zhang G., Li R., Duan L. (2013). Preliminary revision of the posttraumatic growth inventory for military academy students. Chinese Journal of Clinical Psychology.

[B59-behavsci-15-00335] Zhen R., Zhou X. (2020). A study on the influencing factors of anxiety among the general public during the COVID-19 pandemic. Applied Psychology.

[B60-behavsci-15-00335] Zhong Y., Tang P. F., Fan J. R., Luan J. (2021). Influence of online social support on the public’s belief in overcoming COVID-19. Information Processing and Management.

[B61-behavsci-15-00335] Zhou H., Long L. (2004). Statistical tests and control methods for common method bias. Advances in Psychological Science.

[B62-behavsci-15-00335] Zhou X., An Y., Wu X., Chen H., Long C. (2014). The impact of gratitude on posttraumatic growth among middle school students three and a half years after the Wenchuan earthquake: The mediating role of social support. Psychological Development and Education.

[B63-behavsci-15-00335] Zhou X., Wu X., Wang W., Tian Y. (2017a). The impact of social support on adolescent posttraumatic growth: The mediating roles of state hope and positive reappraisal. Psychological Development and Education.

[B64-behavsci-15-00335] Zhou X., Wu X., Wang W., Tian Y. (2017b). The relationship between social support, posttraumatic stress disorder, and posttraumatic growth: A longitudinal study of elementary school students after the Ya’an earthquake. Acta Psychologica Sinica.

[B66-behavsci-15-00335] Zhou X., Wu X., Wang W., Tian Y. (2019). The relationship between social support and posttraumatic growth among adolescents 8.5 years after the Wenchuan earthquake: The mediating roles of self-efficacy and self-esteem. Psychological Development and Education.

[B65-behavsci-15-00335] Zhou X., Wu X. C., Zhen R. (2017c). Understanding the relationship between social support and posttraumatic stress disorder/posttraumatic growth among adolescents after Ya’an earthquake: The role of emotion regulation. Psychological Trauma.

[B67-behavsci-15-00335] Zhu X., Luo F., Yao S. (2007). The psychometric properties of the Chinese version of the Cognitive Emotion Regulation Questionnaire (CERQ-C). Chinese Journal of Clinical Psychology.

